# *Ormosia viridiflora*, a new species of Fabaceae from Guangxi, China

**DOI:** 10.3897/phytokeys.280.214416

**Published:** 2026-10-22

**Authors:** Chunyu Zou, Zhaocen Lu, Suyun Nong, Yan Liu

**Affiliations:** 1 Guangxi Key Laboratory of Plant Conservation and Restoration Ecology in Karst Terrain, Guangxi Institute of Botany, Guangxi Zhuang Autonomous Region and Chinese Academy of Sciences, Guilin, 541006, Guangxi, China Guangxi Key Laboratory of Plant Conservation and Restoration Ecology in Karst Terrain, Guangxi Institute of Botany, Guangxi Zhuang Autonomous Region and Chinese Academy of Sciences Guilin China

**Keywords:** Morphology, Papilionoideae, pollen, taxonomy

## Abstract

A new species, *Ormosia viridiflora* C.Y. Zou & Yan Liu, from Guangxi, China, is described and illustrated. It differs from its congeners by the following combination of characters: shrubby habit; 5–11 leaflets with caudate apices; glabrous adaxial and subglabrous abaxial leaflet surfaces; tubular calyx; pods with leathery valves and inconspicuous transverse septa; pseudo-peltate standard petal (with basal auricles sheathing the claw apex); and five stamens. Palynological and leaf epidermal features are also documented. A preliminary IUCN assessment of Critically Endangered (CR) is proposed.

## Introduction

*Ormosia* Jack. is a genus of papilionoid legumes (Fabaceae, Papilionoideae) with a pantropical and subtropical distribution, comprising approximately 130–150 species of trees and shrubs (Torke et al. 2022; Guevara-Andino et al. 2025). As one of the early-diverging groups within the subfamily Papilionoideae (Cardoso et al. 2012b, 2013), *Ormosia* is characterized by its imparipinnate leaves, papilionaceous flowers usually with 10 free stamens, and distinctive seeds with a hard, often red or black testa (Rudd 1965; Chang 1984a; Cardoso and Queiroz 2010). In China, the genus is represented by approximately 35–37 species, predominantly distributed in the southern provinces of Guangxi, Guangdong, Yunnan, and Hainan (Niu et al. 2023; Zhang et al. 2026).

Historically, the classification of *Ormosia* has relied heavily on vegetative characters, particularly leaf morphology (Niu 2015). However, recent studies have emphasized the critical taxonomic importance of reproductive features (Niu 2015). A comprehensive taxonomic study of Chinese *Ormosia* has proposed that floral morphology and fruit characters hold greater diagnostic value than vegetative traits, with attributes such as corolla shape, the ratio of calyx tooth length to tube length, pedicel length, and fruit characters providing essential criteria for species delimitation (Niu 2015). These findings offer useful perspectives for species delimitation, although they have not fundamentally changed the taxonomic concepts of the Chinese species. Furthermore, pollen micromorphology—including exine ornamentation and aperture type—has also been suggested to possess taxonomic significance within the genus (Niu 2015; Zhang et al. 2022). Despite these advances, the infrageneric classification of *Ormosia* remains problematic. This is not merely a matter of insufficient field collections or the limited number of characters used in previous systems; rather, the major challenge lies in obtaining a robust phylogenetic framework. Although Sanger-based molecular studies, particularly that of Torke et al. (2022), have made substantial progress, much greater resolution at the genomic level is still needed before a stable and well-supported infrageneric classification can be proposed. These limitations underscore the need for continued taxonomic exploration.

While a comprehensive infrageneric classification remains elusive, recent molecular phylogenetic work has substantially improved the understanding of evolutionary relationships within *Ormosia*. The most extensively sampled phylogeny to date, based on nuclear ITS and plastid markers from 82 species, recovered strong support for the monophyly of the genus and revealed that all sampled Asian species fall into two comparably sized clades, one of which is sister to the clade containing all sampled New World species (Torke et al. 2022; Guevara-Andino et al. 2025). This finding has direct implications for the taxonomy of Chinese species: several widely distributed taxa, including *O. emarginata* and *O. semicastrata*, were recovered in different Old World clades despite their historical placement in the same infrageneric groups. The phylogenetic non-monophyly of traditional morphology-based sections underscores the need for a thorough reassessment of the infrageneric classification of Chinese *Ormosia*. Niu (2015) likewise recognized that the current system, which relies heavily on fruit and seed characters, does not reflect evolutionary relationships and proposed that floral and vegetative traits should be re-evaluated in a phylogenetic context. These findings reinforce the rationale for emphasizing reproductive characters in the present study.

The taxonomic history of Chinese *Ormosia* extends back to Bentham’s early groupings and was formalized in Merrill and Chen’s (1943) treatment of the Chinese and Indo-Chinese species, which recognized 34 species and 15 series based largely on pod and seed morphology. This foundational work established the morphological framework that subsequent authors refined, including Yakovlev’s (1971–1976) controversial split of the genus into six separate genera and Chang Rui-Hua’s (Chang 1984a, 1984b) revision recognizing 35 species in three sections and six series. The treatment in Flora of China (Sun and Vincent 2010) synthesized these earlier efforts, accepting 35 species and providing the most comprehensive account of Chinese taxa to date. However, as noted by Niu (2015) and corroborated by observations in the present study, these systems remain constrained by reliance on a limited set of morphological characters, particularly dry pod and seed features, which exhibit considerable homoplasy. This historical context clarifies both the basis for recognizing *O. viridiflora* as distinct and the need for additional diagnostic characters—such as the floral and fresh pod features emphasized in the present study—to resolve species boundaries within the genus.

Recent years have witnessed the discovery of several new *Ormosia* species from previously underexplored regions, including *O. neillii* from Ecuador (Guevara-Andino et al. 2025), *O. corcovada* from Colombia (Herrera-Palma et al. 2022), and *O. yangjiangensis* and *O. taishanensis* from southern China (Zhang et al. 2026). These findings suggest that the species diversity of *Ormosia* is still far from fully understood, particularly in areas with complex topography and high habitat heterogeneity.

During botanical surveys in Guangxi, an unusual *Ormosia* species was encountered that could not be assigned to any known taxon. Based on overall morphological similarity and previous taxonomic treatments (Chang 1984a), the new species is most closely comparable to *O. saxatilis* and *O. microphylla*. A detailed morphological examination using newly collected material, especially comparisons between fresh and dried fruits, with particular attention to controlling for the effects of drying on specimen appearance, revealed consistent and significant differences from these two species, particularly in growth habit, floral scent, and fruit drying behavior. Although fruits and seeds have traditionally been the most important characters in the taxonomy of *Ormosia*, floral morphology at anthesis has been largely neglected. Given the taxonomic significance of floral morphology in the genus, these distinctive features support the recognition of the species as new to science. The species is therefore described and illustrated here, with detailed morphological comparisons and a discussion of the implications of its floral characters for species delimitation within the genus.

## Materials and methods

The taxonomic decision to recognize the new species was made in the context of a broader study of Chinese *Ormosia*. Over the past 3 years, extensive field surveys were conducted across the distribution range of the genus in China, including visits to the type localities of many species; as a result, floral and fruit information is now available for approximately half of the Chinese species. Specimens from major Chinese herbaria (e.g., IBK, IBSC, KUN, and PE), including type specimens (some examined as digital images), were also examined, and the protologues of all Chinese names as well as relevant literature on Asian *Ormosia* (e.g., Merrill and Chen 1943; Niyomdham 1980; Chang 1984a, 1984b; Torke et al. 2022) were consulted. The measurements and descriptions of the new species were based on both fresh and dried herbarium specimens collected from the wild. Measurements were performed with a ruler, and small plant parts were observed and measured using a scanning electron microscope (ZEISS EVO18). To discern morphological distinctions from related species, particular attention was paid to floral characters, and comparisons were made with *O. saxatilis* and *O. microphylla* using newly collected material, including fresh and dried fruits. The type specimens were deposited at IBK, PE, KUN, IBSC, and HITBC.

## Taxonomy

### Ormosia viridiflora

Taxon classificationPlantaeDipteraLimoniidae

C.Y.Zou & Yan Liu sp. nov.

A28B7D17-5588-5F48-9E63-1A47ADEC3CB6

urn:lsid:ipni.org:names:77422913-1

Figs 1, 2

#### Type.

China • Guangxi, Liuzhou City, Rongan County, Banlan Town, in the forest, 706 m elev., 27 Mar. 2026, *C.Y. Zou, Y. Qing & Y. Liu ZCY6622* (holotype: IBK!; isotype: HITBC!, IBSC!, KUN!, PE!).

**Figure 1.**
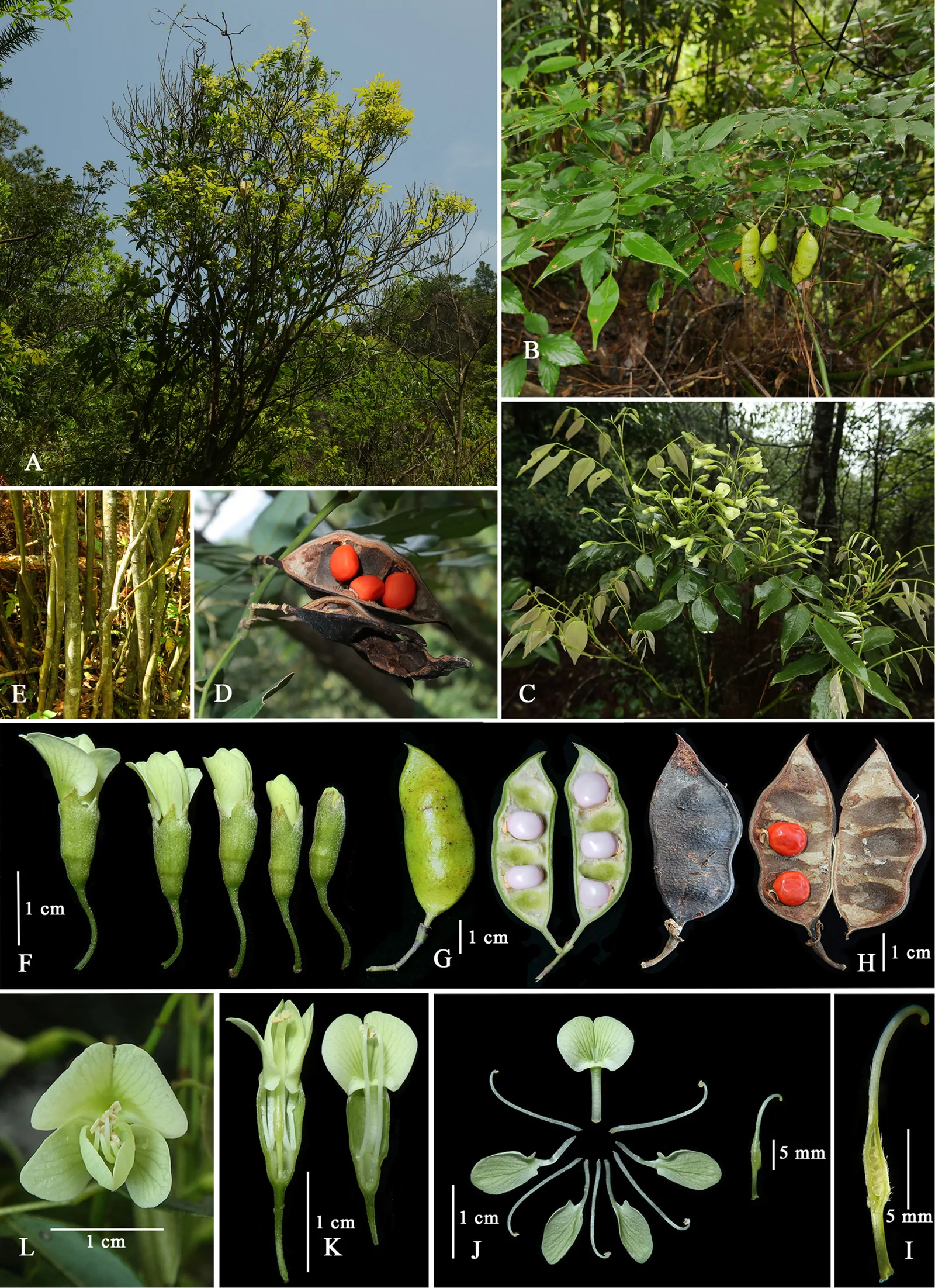
*Ormosia viridiflora*. **A**. Habit; **B**. Fruiting branches; **C**. Flowering branch, mature and fresh; **D**. Fruiting branches, dry and dehisced; **E**. Caespitose trunk; **F**. Flowers at different developmental stages; **G**. Fresh fruit and fruit in transverse section; **H**. Dry fruit and fruit in transverse section; **I**. Longitudinal section of ovary; **J**. Dissected flower; **K**. Longitudinal section of flower; **L**. Frontal view of flower. **A, B**. Photographed by Ming Su; **C**. Photographed by Ping Yang; all other photographs by Chun-Yu Zou.

**Figure 2.**
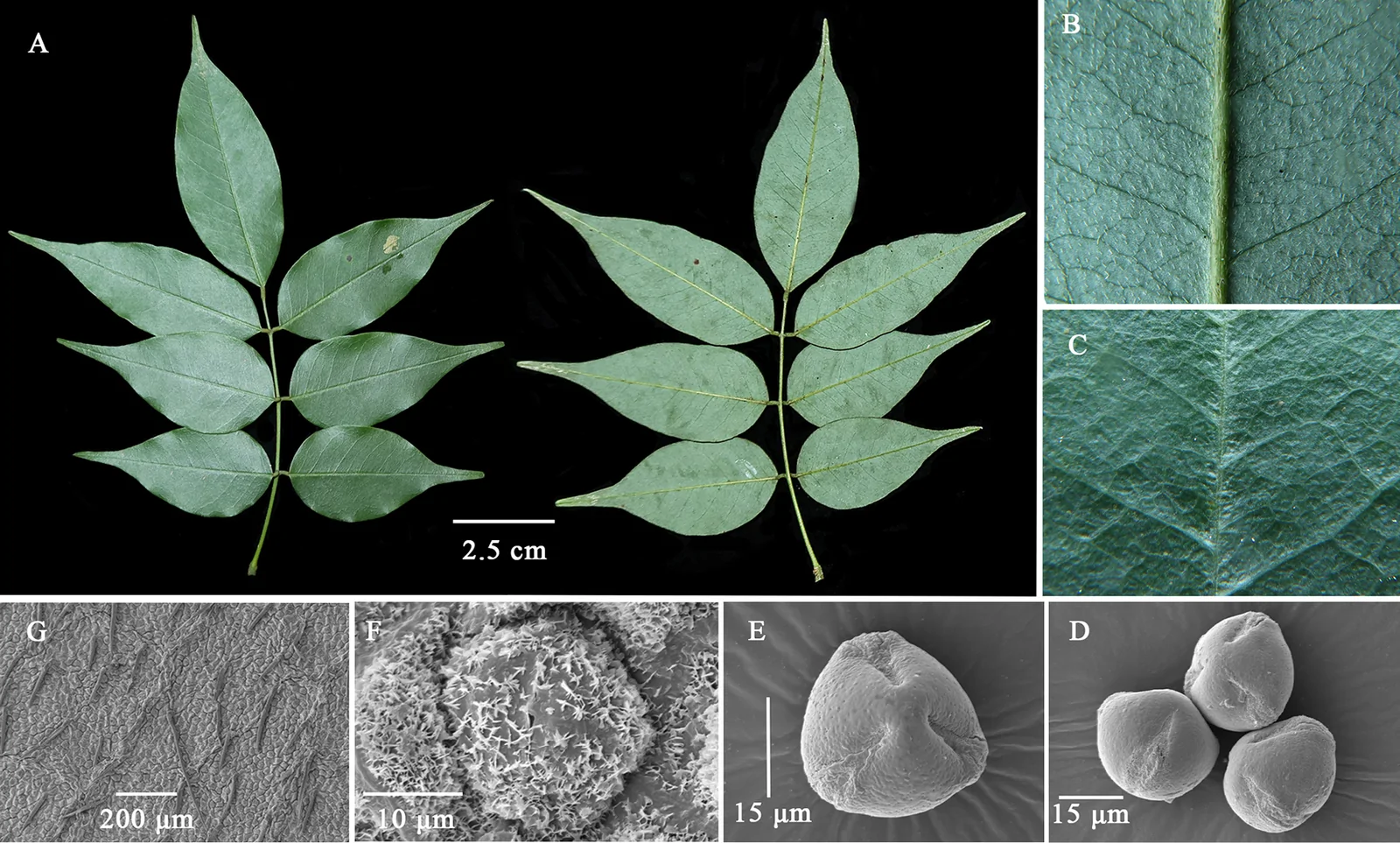
Leaf morphology and leaf and pollen micromorphology of *Ormosia viridiflora*. **A**. Adaxial and abaxial views of a fresh leaf; **B**. Leaf adaxial surface under a 10× magnifying glass; **C**. Leaf abaxial surface under a 10× magnifying glass; **D**. Apocolpium ornamentation of pollen grains; **E**. Mesocolpium ornamentation of a pollen grain; **F**. Rosette wax crystals; **G**. Leaf abaxial indumentum morphology. **A–C**. Photographed by Chun-Yu Zou; **D–G**. Photographed by Yu-Jing Wei (SEM).

#### Diagnosis.

*Ormosia viridiflora* differs from *O. microphylla* and *O. saxatilis* by its shrubby habit (vs. tall trees); leaflets 5–11, apex caudate (vs. 11–17 or 15–23, apex acuminate); leaflets glabrous adaxially and subglabrous abaxially (vs. pubescent or densely hairy); tubular calyx (vs. campanulate); pods with leathery valves and inconspicuous transverse septae (vs. thickly woody and transversely septate valves in *O. microphylla*, thinly woody and transversely septate valves in *O. saxatilis*); standard petal pseudopeltate (vs. neither peltate nor pseudopeltate); and five fertile stamens without staminodes (vs. 10 stamens).

#### Description.

Shrub, 2–3(–4) m tall; trunk with outer bark green. Current-year branchlets, petioles, leaf rachises, and pulvinules densely white-tomentose, becoming glabrous in fruit. Stipules not seen. Leaves 6–18 cm long, imparipinnate, 5–11-foliolate; pulvinus 2–4 × 2–3 mm, terete; petiole 1.7–3.0 cm long; rachis 2.5–7 cm long, interfoliolar segments 1–2 cm long; stipels absent; leaflets opposite, pulvinules 2.5–3.5× ca. 1 mm, terete; blades 2.8–7.5 × 1.5–3 cm, thinly leathery, oblong-elliptic to broadly elliptic, base rounded or obtuse, apex caudate, adaxially green and glabrous, abaxially pale and appressed white-pubescent, with rosette-like epicuticular wax crystals on the abaxial surface; midvein abaxially prominent, secondary veins inconspicuous. Inflorescence terminal or axillary in upper leaves, a lax panicle or raceme, 5–10 cm long, white-pubescent; pedicels slender, 1.5–1.9 mm long, bearing a single minute bracteole at base, ca. 1 mm long; flower buds 12–15 × 3–4 mm, oblong. Flowers 1.7–1.8 cm long, papilionaceous; calyx tubular, tube 7–9 mm long, ca. 4 mm wide, abaxially densely white-puberulent, adaxially green, lobes triangular, 3–4.5 mm long, unequal; petals light green, free, glabrous, long clawed at base; standard, 8–9 × 8–10 mm, with dark green stripes at base, suborbicular, peltate, apex emarginate, claw 6–7 mm long; wing 8–8.5 × 3.5–4.0 mm oblong, claw 5–6 mm long; keel blades oblong, 7–8 × 3–4 mm, claw 5–6 mm long; stamens five, subequal, filaments 11–13 mm long, free, glabrous, apically curved, anthers basifixed, elliptic; pollen grains oblate-spheroidal, oblong in equatorial view, 3-lobed circular in polar view, tricolporate, 23.5–26.5 × 21.5–24.0 μm, *P/E* = 1.09; colpi fusiform, endoapertures transversely elongated; exine microreticulate; pistil 15–18 mm long, ovary 4–5× ca. 1 mm, oblong in outline, laterally compressed, sparsely pilose, stipe 4–5 mm long, ovules 1–5; style ca. 6 mm long, glabrous, apically curved, with a bilobed stigma. Pods 3–5.5 × 1.3–2.4 cm, dehiscent along both sutures, suborbicular to ovate when 1-seeded, oblong-elliptic when more than 2- or more-seeded, apex cuspidate, glabrous at maturity; valves leathery, with inconspicuous transverse septa. Seeds 1–5, 8–10 × 8–9.5 mm, light red to dark red, ovate to suborbicular in outline, slightly compressed; seed coat is hard; hilum elliptic, 2.5–3 × 2.3–2.8 mm.

#### Phenology.

*Ormosia viridiflora* is known to flower from late March to early April and fruit in September.

#### Etymology.

The specific epithet *viridiflora* refers to the light green color of both the corolla and the calyx.

#### Distribution and habitat.

This new species is known only from the type locality, i.e., Rongan County, Guangxi, China. It grows in thickets with species such as *Rhododendron latoucheae* Franch., *Daphniphyllum oldhamii* (Hemsl.) K. Rosenthal, and *Pinus massoniana* Lamb. It grows within evergreen mixed forests at an elevation of 600–750 m, on acidic mountain yellow soil.

#### Additional specimens examined.

China • Guangxi, Liuzhou City, Rongan County, Banlan Town, growing in thickets, 612 m elev., 4 Sept. 2019, *Rong’an County Expedition 450224190904046LY*(IBK!), same locality, 10 Aug. 2026, *C.Y. Zou ZCY 6815*(IBK!).

#### Conservation status.

Since the species was first collected in the field in 2019, no additional populations have been found outside the type locality, and only a single subpopulation has been recorded. The extent of occurrence (EOO) and area of occupancy (AOO) remain unclear due to the lack of comprehensive field surveys; however, the species is evidently restricted to a single location. The type locality is situated at the edge of a plantation and is currently subject to direct human disturbance. The extremely narrow distribution and tiny population size imply a high risk of extinction, with an inferred continuing decline in habitat quality (criterion iii). According to the Guidelines for Using the IUCN Red List Categories and Criteria (IUCN Standards and Petitions Committee 2022), *Ormosia viridiflora* meets the thresholds for criteria B1ab(iii)+2ab(iii), C2a(i), and D. Criterion D (fewer than 50 mature individuals) alone qualifies this species as Critically Endangered (CR), while criteria B and C may indicate Endangered (EN) or Vulnerable (VU), depending on precise EOO/AOO values. A tentative assessment of CR is therefore recommended, pending further field investigations. Effective conservation measures for this species remain a priority for future research.

#### Similar species and notes.

**Figure 3.**
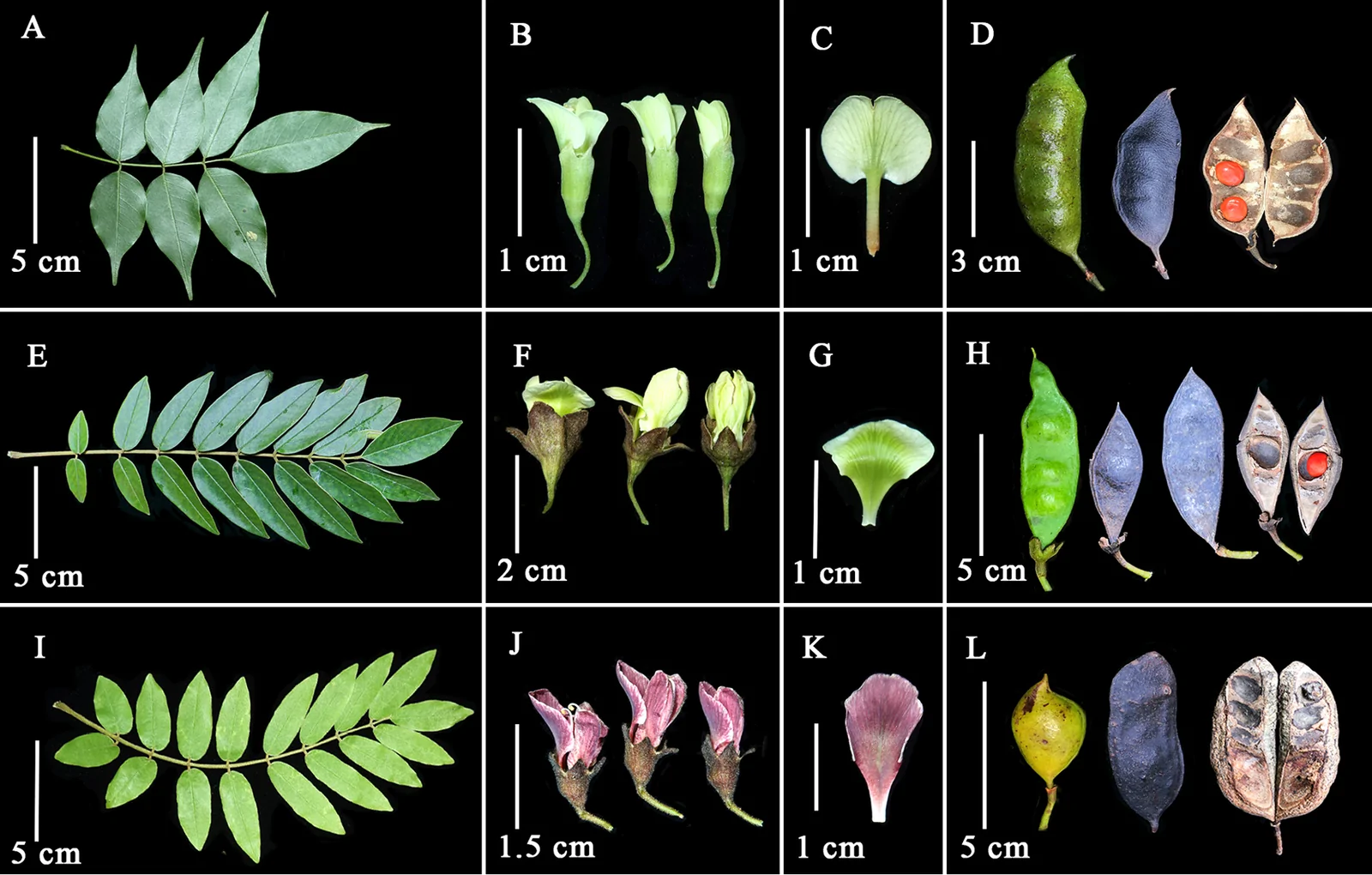
Comparative morphology of *Ormosia viridiflora* (**A–D**), *O. saxatilis* (**E–H**), and *O. microphylla* (**I–L**). **A, E, I**. Leaves; **B, F, J**. Flowers; **C, G, K**. Standards; **D, H, L**. Fresh and dried fruits.

*Ormosia viridiflora* compares most closely in morphology with *O. saxatilis* and *O. microphylla* in floral and fruit characters, but it can be distinguished from both by a combination of vegetative and reproductive characters. The leaf morphology and pollen characters support the placement of *O. viridiflora* in *Ormosia*. Pollen grains are oblate-spheroidal, oblong in equatorial view, and 3-lobed circular in polar view, tricolporate, 23.5–26.5 × 21.5–24.0 µm (medium-sized, cf. Erdtman 1969), with *P/E* = 1.09; colpi fusiform, endoapertures transversely elongated; exine microreticulate. These pollen features, together with the leaf indumentum, are consistent with the genus. The rosette-like wax crystals are apparently previously unrecorded in *Ormosia* and may be diagnostic for the new species, while the sparsely pubescent abaxial leaf surface contrasts with the pubescent or densely hairy leaflets of *O. microphylla* and *O. saxatilis*. Within Asian *Ormosia*, these three species appear to form a morphological assemblage by sharing coincident flowering periods; glabrous, transversely septate pods (septa inconspicuous in *O. viridiflora*); mature fresh pods that are swollen and, upon drying on the tree, turn black and decrease in thickness by nearly one-quarter to one-third; and a red, hard, difficult-to-remove seed coat. This combination of characters is not known in other Asian congeners. The new species is a shrub, reaching ca. 4 m in height with a DBH of 1.5–3 cm, whereas *O. saxatilis* and *O. microphylla* are typically tall trees. Its flowers lack obvious fragrance, while *O. microphylla* produces a strong fragrance during anthesis (fragrance data unavailable for *O. saxatilis*). In fruit characters, mature fresh fruits of *O. viridiflora* and *O. microphylla* are both swollen, but those of *O. viridiflora* turn black and become thinner upon natural drying on the tree, whereas those of *O. microphylla* do not exhibit this change; fruits of *O. saxatilis* remain flattened from maturity through to complete drying. Pollen characters are otherwise conserved within the genus, and comparable data for *O. microphylla* and *O. saxatilis* are lacking; they therefore do not independently resolve the affinities of *O. viridiflora*. Its proposed relationship with *O. microphylla* and *O. saxatilis* remains based mainly on gross morphology and fruit characters and requires phylogenetic testing. A detailed morphological comparison among the three species is provided in Table 1 and Fig. 3. The five fertile stamens, which also occur in *O. semicastrata*, might suggest a relationship with *O. semicastrata* or may represent an independent reduction; this character may be homoplastic within *Ormosia*. In *O. viridiflora*, no staminodes were observed in dissections from 2025 and 2026, although one photographic record shows a possible staminode of undetermined number. In *O. semicastrata*, flowers with five fertile stamens and no staminodes are common, but 1–3 staminodes also occur. Thus, staminodes in these species appear highly reduced and variable, being absent or difficult to observe. Five fertile stamens are rare in Asian *Ormosia*. In tropical American papilionoids, five fertile stamens without staminodes also occur in the monotypic *Guianodendron* (Schütz Rodrigues and Tozzi 2006; Cardoso et al. 2012a). A phylogenetic analysis including Asian *Ormosia*, especially *O. saxatilis*, *O. microphylla*, and *O. semicastrata*, is planned to test these hypotheses.

**Table 1.** Morphological comparison of *Ormosia viridiflora*, *O. saxatilis*, and *O. microphylla*.

**Characters**	***Ormosia viridiflora***	***O. saxatilis***	***O. microphylla***
**Habit**	Shrubby	Arboreal	Arboreal
**Leaflets**	5–11-foliolate, glabrous or subglabrous, apex caudate	15–23-foliolate, densely yellowish-brown tomentose, apex acuminate	11–17-foliolate, densely yellowish-brown pubescent, apex acuminate
**Calyx**	Tubular, white-puberulent	Campanulate, densely yellowish-brown tomentose	Campanulate, densely blackish-brown tomentose
**Flower color**	Light green	Light green	Purple or light purple
**Standards**	Suborbicular, claw pseudopeltate, claw 6–7 mm long	Fan-shaped, claw neither peltate nor pseudopeltate, claw 3.5–4.0 mm long	Fan-shaped, claw neither peltate nor pseudopeltate, claw 3.3–3.7 mm long
**Stamens**	5	10	10
**Legumes**	Turgid, without rib, thinly leathery ca. 1 mm	Compressed, with rib, thinly woody, ca. 1 mm	Turgid, with rib, thickly woody, ca. 2–3 mm
**Seeds**	1–5	1–6	1–4

## Supplementary Material

XML Treatment for Ormosia viridiflora

## Additional information

### Artificial Intelligence (AI) use

The authors accept full responsibility for the content of the manuscript, including the disclosure of any use of AI.

No AI tools were used in the preparation of this manuscript.

### Author ORCIDs

C. Zou https://orcid.org/0000-0001-6004-6551

Z. Lu https://orcid.org/0000-0002-1725-0247

S. Nong https://orcid.org/0000-0001-5622-815X

Y. Liu https://orcid.org/0000-0001-8931-9656

### Data availability

All of the data that support the findings of this study are available in the main text.
